# Can chiropractors contribute to work disability prevention through sickness absence management for musculoskeletal disorders? - a comparative qualitative case study in the Scandinavian context

**DOI:** 10.1186/s12998-018-0184-0

**Published:** 2018-04-26

**Authors:** Mette Jensen Stochkendahl, Ole Kristoffer Larsen, Casper Glissmann Nim, Iben Axén, Julia Haraldsson, Ole Christian Kvammen, Corrie Myburgh

**Affiliations:** 1 Nordic Institute of Chiropractic and Clinical Biomechanics, Campusvej 55, DK-5230 Odense M, Denmark; 2 Department of Sports Science and Clinical Biomechanics, University of Southern Denmark, Campusvej 55, DK-5230 Odense M, Denmark; 3 Horten, Norway; 4 Unit of Intervention and Implementation Research for Worker Health, Institute of Environmental Medicine, Karolinska Institutet, Stockholm, Sweden; 5 Falkenberg, Sweden; 6 Sandefjord, Norway; 7 Institute of Health and Society, University of Oslo, Kirkeveien 166, Frederik Holsts hus, 0450 Oslo, Norway

**Keywords:** Chiropractic, Policy, Work disability prevention, Sickness absence, Qualitative, Interview

## Abstract

**Background:**

Despite extensive publication of clinical guidelines on how to manage musculoskeletal pain and back pain in particular, these efforts have not significantly translated into decreases in work disability due to musculoskeletal pain. Previous studies have indicated a potential for better outcomes by formalized, early referral to allied healthcare providers familiar with occupational health issues. Instances where allied healthcare providers of comparable professional characteristics, but with differing practice parameters, can highlight important social and organisational strategies useful for informing policy and practice. Currently, Norwegian chiropractors have legislated sickness certification rights, whereas their Danish and Swedish counterparts do not. Against the backdrop of legislative variation, we described, compared and contrasted the views and experiences of Scandinavian chiropractors engaging in work disability prevention and sickness absence management.

**Methods:**

This study was embedded in a two-phased, sequential exploratory mixed-methods design. In a comparative qualitative case study design, we explored the experience of chiropractors regarding sickness absence management drawn from face-to-face, semi-structured interviews. We subsequently coded and thematically restructured their experiences and perceptions.

**Results:**

Twelve interviews were conducted. Thematically, chiropractors’ capacity to support patients in sickness absence management revolved around four key issues: issues of legislation and politics; the rationale for being a sickness absence management partner; whether an integrated sickness absence management pathway existed/could be created; and finally, the barriers to service provision for sickness absence management.

**Conclusion:**

Allied health providers, in this instance chiropractors, with patient management expertise can fulfil a key role in sickness absence management and by extension work disability prevention when these practices are legislatively supported. In cases where these practices occur informally, however, practitioners face systemic-related issues and professional self-image challenges that tend to hamper them in fulfilling a more integrated role as providers of work disability prevention practices.

## Background

Musculoskeletal pain is a major cause of work disability with enormous socioeconomic consequences. Back pain-related disorders alone are costly and responsible for up to one quarter of days off work in European countries like Sweden [1] and Denmark [2], and in Norway four out of ten sickness certifications are based on a musculoskeletal diagnosis [3].

For patients with musculoskeletal pain or other work-related problems, general practitioners (GPs) are the traditional gatekeepers to workers’ compensation through sickness certification in the majority of European countries, but studies from the UK and Scandinavia have indicated that GPs question the relevance of work-related issues to their primary healthcare provider role [4–8]. Restraints in terms of time and resources and of lack of knowledge around judging capacity to work have been identified as major barriers for GPs to engage with social workers and workplaces [9, 10]. Furthermore, some GPs would prefer not to be part of the sickness certification system, suggesting the alternative of an authoritative individual to whom they could refer patients [7, 11]. This leaves a missed potential for relevant workplace assessments, and for engaging in dialogue with the patient and the employer regarding work accommodations. Further, to provide evidence-based guidance to encourage early self-management and a continuation, or early resumption of work activities [12], such a dialogue is necessary. The GPs’ solitary role in sickness certification may also result in lack of collaboration between clinicians and other stakeholders, which has been identified as detrimental for a positive return to work outcome [13].

The use of allied healthcare providers (AHP), like physiotherapists, chiropractors and manual therapists, within the field of musculoskeletal pain is gaining popularity amongst patients, especially in the working population [14]. AHP are also more often sought out as the first points of contact and principal providers of healthcare for individuals with musculoskeletal conditions [15, 16]. This poses a challenge for the continuity and coordination of care when sick leave certification is required as many of these patients may not see another practitioner about their back pain [17], while others may also consult their GP. In the context of work, the integration of healthcare professionals may be even more challenging as outcomes are not merely dependent on high quality healthcare, but also the collaboration of multiple stakeholders inside and outside the healthcare sector and the workplace [14].

As the population ages and the current health reforms focus on shifting secondary care services into the community, demands on GPs and primary healthcare continue to rise [18, 19]. Despite the publication of clinical guidelines on how to manage musculoskeletal pain in general and back pain in particular, these efforts have not significantly translated into decreases in work disability due to musculoskeletal pain as evident by the continuously high costs to society. With the substantial cost implications of work disabilities for national economies, there is a need for improvement in the way healthcare systems and their actors incorporate work disability prevention (WDP) in their service for individuals with musculoskeletal conditions. Moreover, there is a need for improving the communication and collaboration between the healthcare actors, employees and workplaces. Previous studies have indicated a potential for better work disability outcomes by formalized, early referral patterns to AHPs familiar with occupational health issues [20–22]. Therefore, one potential strategy could be to integrate WDP in the model of care provided by AHPs [20–22] for patients with musculoskeletal disorders.

### Chiropractors’ sickness absence management practices across Scandinavia

Occupational groups operating within similar social contexts, but with varied legislated practice parameters, provide an opportunity to observe the impact of systematic variation [23]. More specifically, highlighting the social sequelae of different sick leave management practices is useful for informing policy and practice [24].

Chiropractic is a growing musculoskeletal health profession in Norway, Denmark, and Sweden. It is *concerned with the diagnosis, treatment and prevention of mechanical disorders of the musculoskeletal system.* Members of the respective national chiropractic associations hold a 4- or 5-years Master’s degree in musculoskeletal health, which, when followed by a 1-year internship, qualifies for the respective national board of health certifications as independent healthcare providers. In all three Scandinavian countries, chiropractors function as first points of contact for patients with musculoskeletal disorders, but under different regulations and levels of integration in the welfare systems. In Sweden, chiropractors are largely private musculoskeletal practitioners outside the national health service with limited integration into the national healthcare system, while in Denmark and Norway, the chiropractors work inside the respective national health services as AHPs.

In Norway, chiropractors and manual therapists (i.e. physiotherapists with a degree at the masters level) received authorization to issue sickness certifications for 0–8 weeks in 2006, and since 2008, for 0–12 weeks [25]. However, in the two other Scandinavian countries, Denmark and Sweden, no such regulation currently exists (see Table 1). In Denmark, in 2009, the traditional sickness certification was replaced by a fitness for work certificate (“fit note”), which describes how the patient’s condition influences their work situation and work role functioning. The employee, employer and GP all contribute information to the certificate, and the purpose of the fit note is to facilitate return to work. Thus, the GP no longer must sanction sickness absence in order for the employee to receive benefits. A detailed description of the three countries’ legislations and regulations is presented in Table 1.

**Table 1:** Key facts relating to the chiropractors’ role in sickness absence management across Norway, Denmark, and Sweden

	Denmark	Norway	Sweden
Healthcare system			
Public funding (tax-funded)	Yes	Yes	Yes
Sickness absence legislation			
Certification required	No	Yes, if more than 3 days	Yes, if more than 7 days
Sickness benefits paid by employer^a^	30 days	16 days	2–14 days
Sickness benefits paid by social services^a^	> 30 days- 23 weeks	> 16 days −1 year	> 14 days – 1 year
Chiropractors			
Sick leave certification rights	Not applicable (“fit-note”)	Yes (0–12 weeks)	No
Referral rights to imaging (e.g., x-ray or MRI) and medical specialists	Yes, partially (musculoskeletal related)	Yes, partially (musculoskeletal related)	No
Practice forms	Allied healthcare within the national healthcare system	Allied healthcare within the national healthcare system	Private musculoskeletal healthcare providers
Regulated under the national health services	Yes	Yes	Largely outside^b^
Privately owned clinics	Yes	Yes	Yes
Patient fee for service	Partial	Partial	Full (private) and partial (public)^b^

^a^Presented are overall rules. Specifics may apply\

^b^Approximately 20% of clinics have agreements under the national health services

In the context of changing healthcare policies and organisational structures, there is an increasing need for evaluations of the impact of role extensions, and the potential barriers and facilitators for implementation of such a change.

The objectives of this study were to:

1. Describe the experiences of chiropractors engaging in sickness absence management (SAM).
2. Compare and contrast chiropractors’ integration of SAM in their model of care in a context with legislated sickness certification rights (Norway) and in two contexts without sickness certification rights (Sweden and Denmark).

## Methods

This study formed part of a two-phased sequential, exploratory mixed-methods design (the results of the quantitative phase will be reported separately) [26].

Using a postpositivistic lens, a comparative qualitative case study [27] was conceptualized in order to analyse and understand detailed and in-depth descriptions about the experience of Swedish, Norwegian and Danish chiropractors regarding SAM.

### Sampling and recruitment

During the period of June 2015 to March 2016, we purposively sampled chiropractors across the three countries with experiences regarding SAM who were willing to share these [28]. Specifically, we recruited chiropractors with a recent experience in managing patients with work disability and were seeking a variety of practice types (solo/group/multidisciplinary), location (country), and “other interests” (additional occupation/board membership). Chiropractors who were identified by a project gatekeeper from the research group’s network were invited by email. Further, chiropractors were invited via a snowball technique through the chiropractors’ networks [29].

### Interviews

An interview guide with prompts was developed where the team drew on their experience of work disability programs as researchers and as clinicians, and on recent research on the topic. Face-to-face, semi-structured interviews were conducted where the chiropractors were asked to draw on examples of cases from their own experience [28]. A rolling interview schedule was used. That is, questions were modified over the course of the interviews to ensure that responses to key cross-case topics were elicited (see Table 2). In the interviews, participants were asked to talk about their personal experience and level of involvement in SAM of patients with musculoskeletal pain. The interviews were designed to map out the chiropractors’ general experience in clinical practice. The participants were then asked to share their perception of their current role and competencies, and to talk about the support and training they would need to better assist an individual patient in these matters.

**Table 2:** Topics discussed and examples of trigger questions during the interviews

Topics discussed	Examples of questions
Current role in SAM	• How are you currently involved in managing sickness absence of your patients? • What is your experience regarding sickness absence of your patients? • In what kind of patients do you typically consider sickness absence? • How often do you certify/advise about sickness absence? • How do you assess the patients regarding SAM? • How do feel equipped to engage in SAM?
Collaboration with stakeholders	• How do you collaborate with the general practitioner/work place/social services regarding SAM?
Barriers and facilitators for SAM	• Which challenges have you met in managing sickness absence? • What do you see as facilitators for managing sickness absence?
Future role	• Given the possibility, which initiatives or collaborations would support you in your work regarding SAM? • How do you envision the future collaboration between the general practitioners and you – who has which role? • How do you think you can contribute to managing sickness absence? - What role would you like to play?

*SAM* Sickness absence management

At the commencement of each interview, demographic data (i.e. gender, age, practice type, and other work functions) were collected. Interviews were conducted in the native language of the participants by one or two out of three interviewers from the research team at locations convenient for the participants. Each interview was audio-recorded and transcribed verbatim by the research team into computer-readable text files. During the interview phase of the project, the researchers documented their reflections about the interviews in a journal. These included notes about informal conversations prior to or after the interviews, as well as other information not captured in an audio transcript.

### Data analysis

The interviews were analysed in the language of the participants. The three Scandinavian languages have similar roots and are understood across the three countries, which enabled coding and interpretation of the transcripts in the original language. Further, the team consisted of three bilingual (fluent in Danish/Norwegian; Danish/Swedish; and Danish/English) team members and one trilingual (fluent in Danish/Norwegian/Swedish) team member. Quotes were translated into English by the team members in combination in a way that transferred content equivalence in translation while maintaining semantic equivalence [30]. The research team’s linguistic skills, and cultural and content knowledge assured the accuracy in translation [31].

Two members of the research team (OKL and CGN) independently coded the transcripts using content analysis before discussing the codes and categories with an experienced qualitative researcher (CM) who provided peer consultation about the qualitative data analysis process [28]. A qualitative data analysis package (NVivo, Version 10, QSR International) was used to organize, code and interpret text data. A fourth investigator (MJS), scrutinised sample transcripts, reviewed the coding scheme and analytical decisions, and developed a thematic map. Through an iterative process using memo sharing and consensus meetings, involving all investigators, data was recoded, code families created, and finally, themes were reviewed for coherence.

In instances where new topics emerged, follow-up questions were e-mailed to the previous participants who were asked to state their experience or perception regarding the newly emerged topics. This data was then incorporated into the data analysis to ensure data saturation (i.e., the point where it was felt that no additional information would be produced by increasing the sample size) [28].

### Ethical considerations

In Sweden, the regional ethics committee evaluated the project and found that the study did not need ethical permission (advisory statement 2016/3:1). In Denmark, the Regional ethics of Southern Denmark gave approval for the study and declared that the study does not fall within the scope of the Medical Research Involving Human Subject Act (§14). Approval for data handling and storage covering both Denmark and Norway under the European Economic Area collaboration was granted from the Danish Data Protection Agency. Prior to the interviews, written and oral information about the study were provided to the participants. Written informed consent was obtained from all participants. All participants were advised that conversations were to be audio or video recorded, and assured of confidentiality and anonymity in reporting of the results.

## Results

Twelve chiropractors participated in the study with interviews lasting between 12 and 65 min with a mean of 44 min. The descriptive participant characteristics of our sample are presented in Table 3, below.

**Table 3:** Participant characteristics (*n* = 12)

	Norway (*n* = 4)	Denmark (n = 4)	Sweden (n = 4)
Gender, male/female	3/1	3/1	2/2
Age, years, mean (range)	47 (42–55)	43 (39–50)	38 (36–42)
Type of practice			
Solo/group/multidisciplinary	1/0/3	1/0/3	0/2/2
Other functions			
Active in research projects	1	2	0
Practice consultant		2	0
National association board member	2	0	1
Method of recruitment			
Gatekeeper/snowballing	2/2	4/0	3/1

Four themes emerged from our analysis: Legislation and politics shape sick leave practice, a rationale for the chiropractor as sickness absence manager, an integrated sick leave management pathway and the emergence of the chiropractor as a sick leave manager.

### Legislation and politics shape sick leave practice

Norwegian and Danish chiropractors appeared mindful of maintaining the traditional gatekeeper role of GPs, as they consistently recognized the importance of informing the GPs regarding sick leave issues. There were, however, a variety of opinions about which patients or situations were best coordinated by the GP and which should be supervised by the chiropractor.

When we talk about long-term sick leave, the GP is perhaps a good basis, also because they, they kind of have the whole package [of knowledge]. (DK4-16)

When it’s related to patients within our area of expertise, then it is us who are in control, it is us who know about the course of treatment and follows the patient closely, and it is us who have the main competencies. (DK2-10)

This related mainly to situations where co-morbidity, such as mental disorder had been identified as well as long duration sickness absences.

By contrast, efforts to contribute to SAM in the Swedish context are characterized by uncertainty.

I write why the patient is here and what has been done and what would be good for the patient in the future. So, in that way, you can say it is a grey area. I do not write a sick leave certification, but they can get a document, which says I [the patient] have actually seen a chiropractor with this problem. And it [the problem] has to be rectified or I need help with this. (S4-6)

Danish chiropractors, the group that occupies the legislative “middle ground”, expressed a degree of uncertainty about where the responsibility lay for SAM. In particular, the group’s perception was that current Danish legislation left most of the responsibility to the patient, but that patients were not always aware of the system. They also frequently received requests for assistance from not only patients, but also their place of work with respect to job modification, but they felt unsure whether this type of activity lay within their scope of practice. This issue is illustrated by the following passage from DK4’s interview:

… a lot of citizens are perhaps downright uncertain about, how is it…who can certify sick leave and who will do it. It’s a bit of a grey area, where people are perhaps a little unsure about how the system works, and maybe it’s also an area, where we as chiropractors are a little afraid to open up in that respect. (DK4-1)

Informal roles are furthermore observable in the Danish context as the patient functions as a messenger and the chiropractor takes on the role of arbitrator, balancing agreeing with the patient about sick leave and the need to “push” the patient back to work to prevent unnecessary absence.

In all three contexts, fee-for-service significantly influences SAM:

We are private, it is an expense for the patient, and there are many who do not consult us because of that. … they rather go to the public healthcare, and then they get the sick certification that way. (S2-4)

This filter results in a situation where chiropractors tend not to see patients with a low socioeconomic status, who in turn, are perceived by the chiropractors to be more complicated to manage.

As a consequence, the incentive to engage in complicated SAM was considered low. This was particularly observed in the Danish context:

But it’s the financial part, because you do not get a dime, and it’s actually quite time consuming…and having to write an employer, it takes a long time. You could easily see one, two, three patients instead. (DK2-4)

For the Norwegian and Swedish chiropractors, sick leave certification rights were perceived as a “seal of approval” of the profession. The rights were perceived as a way of becoming fully integrated into the healthcare system, and a way of changing the scope of practice from (alternative) therapy to being recognized for diagnostic and management skills too.

… then they [the patients] appreciate it [the chiropractors’ sickness certification rights], they also see as a quality stamp that we can do it. (NO2-3)

and

… if you want a card to play, which makes chiropractors someone to trust, someone who is known, and who delivers measurable results… (S3-5)

Mirroring the legal status in the two countries, the Norwegians saw the sick leave certification rights as a final approval, the Swedish struggled to gain recognition and mentioned sick leave certification rights as a means to become integrated into the national healthcare system.

### A rationale for the chiropractor as sickness absence manager

Generally speaking, Danish and Norwegian chiropractors perceived themselves as musculoskeletal specialists due to their university degree and as such, as competent as SAM partners for patients with musculoskeletal problems. They felt comfortable in assessing musculoskeletal function and saw the assessments as integral in routine practice.

I think we have a good basis for doing the evaluation, because we actually know the patients well. And we do it [assessments] already at the first visit. (DK3–18).

However, despite the same level of training, the Swedish chiropractors expressed more hesitation regarding their competencies. According to a Swedish respondent:

If you look at long term sick leave and the level of workability and so on, chiropractors are not a group who are used for those kinds of assessments…. No one is educated in that system, but we refer to the GP then. (S1-4)

Across all three contexts, practitioners cited frequent patient contact as a facilitating element in SAM. They explained how this allowed them to get to know the patients through continuous dialogue and to establish trust. As a Norwegian participant explains, routine contact was also thought to provide the opportunity to optimally monitor progression and adapt plans accordingly:

You can continue one week, maybe two weeks, and then have the dialogue with the patient all the time, during treatments too. So, like that you have more options than when you say “I’ll put you off work for three weeks. See you.” And that’s when they return [the patients]. Instead, you have a continuous dialogue about progress. (NO2-4)

Being able to both handle the patients’ musculoskeletal complaint and certify sickness absence was also perceived by chiropractors from all three countries as a means to prevent chronicity. Across contexts our participants argued that the risk of chronicity was a weakness of the current sick leave management system. By adding a chiropractor, the risk would reduce by shortening the chain of management. As S2 explains:

… a disadvantage or a limitation [of the present system], is for the patient, they have to first seek our help and then they have to go further to seek help at the GP who can give them a sick leave certification, if they assess that as necessary. So, it is both the cost, that they meet two health professionals, when it could be enough with one. (S2-7)

Despite some reluctance to engage in SAM due to lack of financial incentive, Danish chiropractors expressed a sense of obligation toward society, and expressed a moral dilemma between service for the greater good or their own pocket. According to DK1:

It’s really an area that is important also socio-economically, and that’s why I think, at one point, we need to step up to the plate as a profession and give it the prestige that it really has, and say “listen, this means something with respect to us saving tax money.” So that Denmark becomes a cheaper country to live in, rather than saying “we focus on our own game, our own everyday life”, but takes on, perhaps something more like a societal supportive function. (DK1-37)

### An integrated SAM pathway

Norwegian and Danish chiropractors described a spectrum of sick leave-related conditions, ranging from uncomplicated musculoskeletal conditions to more complex cases with various degrees of psychosocial and work-related factors, whereas, the Swedish chiropractors predominantly referred to patients with few apparent psychosocial problems.

Well, it is different, right? An office worker, it might be an arm, shoulder or the neck. If the work regards heavy loads, it might be the lower back, for example. (NO3-19)

And then, of course, there are the ones where stress management and all thoughts of things, where it is psychological and the work environment, or a lot of other stuff... (DK4-5)

Well, the patients visiting me who are in need of sick leave are very, very few…most of the patients we see either have had chronic pain for a long time, but are still working,.., or come in with more acute conditions and get symptom free quite fast. (S2–1).

Further, the Danish and Norwegian chiropractors more often described multifaceted patient-centred and work-related action plans,

And then there are situations where you, ehm, again, you have to look at the whole situation. Are there any other factors in play, their employer, their workability, other than just the musculoskeletal? What is the big picture, and so on? (DK3-17)

whereas, the Swedish chiropractors described a focus on manual therapy as their primary tool.

The chiropractors had a general approach to the patients, where emphasis was laid on functional disabilities rather than diagnoses or pain location.

So really, I look at the function of people, even though they have a lot of pain, but I always ask them “Is it worse when you go to work?”…but it starts to hurt after 2 PM [mimics the patient], well, then perhaps you should ask about a shorter working day, right? Or ask if is possible for you to lay down somewhere to rest, like every other hour. Do you have an adjustable table, can you stand and sit, like. So, it’s dependent on what option people have for adjustments. (DK2-20)

Not only did the chiropractors discuss return-to-work, but often mentioned the importance of the patient staying at work despite some degree of pain or disability.

But if it is a person with a more active profession, which does not include heavy lifting and where it is optional to sit, stand, walk etc., then I recommend them to go to work, because if you stay home, you will become inactive and then you have problems for a longer period compared to if you stay active. (S2-2)

Both Norwegian and Danish chiropractors described how early and timely communication between stakeholders was key to successful return-to-work.

Then I write to the GP and state “this is my opinion” so the GP is informed, so at least there is a common ground, because if we start to say something different, then all of the sudden it becomes difficult. (DK2–18).

The standard method of communication with the GPs was via the official electronic platforms, but many felt that the communication was unidirectional, and requested more information going from the GP to the chiropractors.

They never write anything. (DK2-34)

This is contrasted by the Swedish chiropractors, which for the most part did not have access to electronic communication platforms, and did not communicate directly with GPs.

…as the GP, or as everybody is listed in various places is impossible to have communication with everyone. (S2-10)

The Norwegians also described a unidirectional flow of information to the social services, whereas the Danish had very little contact or communication with the social services.

But, perhaps, I would like more collaboration with NAV [social services]. That there was a closer dialogue basically. (NO4-12)

A noticeable difference between the countries was that the Norwegians positively described how SAM was integral to practice. They used SAM as an integral tool and natural part of the care package that they offered their patients. They described SAM as an additional tool in the clinical tool box that they used if they felt it was relevant, and, therefore, as an add-on to clinical practice. They perceived this extra tool as essential to clinical practice, but also an instrument that lead to higher levels of involvement in their patients’ care, which in turn was perceived as challenging and personally and professionally rewarding.

It’s my consistent, positive experience with sickness certification,…. and if I was to move to another country without these rights, I would feel a little helpless and naked, I think. It would take some getting used to because it is such an important tool, because it is so closely linked to the result of what you do and the process, and how you plan a treatment course. It’s not like we’re just therapists anymore, but there is also the advice part, right? (NO3-17)

This was in contrast to most of the Danish and Swedish chiropractors, who were more hesitant about engaging in SAM. The Danes generally described SAM as a tedious process that they were somewhat reluctant to engage in.

Not if you ask the chiropractors, because the paperwork takes up too much. And that’s where you don’t want to spend your time. It’s, it’s the bureaucratic hassle. (DK3-8)

### The emergence of the chiropractor as a sick leave manager

Functioning in the role of a sick leave manager is, as the previous themes indicate, influenced by the individual practitioner, the profession’s state of practice and the social systems that facilitate day-to-day implementation. Our final theme then, chronicles the experiences of chiropractors across the three case settings where these variables are present or absent.

### The individual practitioner

#### Ambivalence regarding embracing the SAM role (Denmark)

Danish chiropractors expressed some degree of ambivalence regarding embracing the SAM role. It was on one hand perceived as a natural progression of the profession, a responsibility to be taken for the greater good, as an obligation or to honour six years of free university schooling, but on the other hand as bothersome and with no financial incentive.

And that [the chiropractors’ role] will slowly change, and that’s why I think there’ll be a big difference between clinics along the way. How many are willing to venture into this, and how many can’t be bothered, and there will be some who can’t be bothered…. We also know there are some [chiropractors] who won’t see chronic patients. And you can’t say that when you have six years of university schooling. Well, you just can’t. (DK2-26)

### Professional practice

#### Systematised channels of communication (Sweden)

For the Swedes, the lack of systematic channels of communication was an important barrier for communication and involvement.

It is a waste of social resources in some way. That the patient all the time has to contact another authority to….Mostly we tell the patients they should contact the GP and then they make the call themselves. We don’t have direct communication with the GP. (S4-5)

Further, the Swedish chiropractors, perceived sickness certification rights as a direct platform for increasing the general communication with GPs and other healthcare professionals:

S4–11: … [SL rights] would be a good commercial for us. Yes, that would initiate an automatic dialogue.

Interviewer: A dialogue with?

S4–11: Other healthcare professions.

#### Administrative burden and collaboration (Denmark)

Danish chiropractors had reservations regarding engaging in SAM because of the administrative burden associated with SAM. The task was considered time consuming and bureaucratic, and lacking the necessary administrative support systems. Especially regarding the collaboration with the social services, which was considered non-existent, unsatisfactory or even adversarial. The chiropractors also reported how they perceived the case managers to have a specific agenda relating to cutting costs to a minimum.

Well, in respect to the Jobcenter [social services], it’s my experience that if you get hold of a specific case worker there on a specific case, then you can make it work, but otherwise it’s often, I think, that the Jobcenter has its own agenda, at least in the area where I work, where I get most of my patients from, they have an agenda, which is fastest return to work. (DK1-13)

The Danish chiropractors alluded to the notion that to avoid paying for the chiropractors’ services, the social services case managers did not stick to procedure protocols.

Then some jolly caseworker emails me and asks about things in relation to the [confidential] social security number and this and that in an unencrypted email. Then sometimes I write them to please don’t include full social security numbers in an open email. And with respect to the actual case, it would be better if we communicated via Status [secure, encrypted communication platform] or similar. (DK1-40)

#### Professional self-image and adaptation in thinking (all)

A salient issue in all three countries is the transition in self-image and adaptation in thinking and behaviour from chiropractors being manual therapists or even alternative care providers to becoming fully integrated members in the primary healthcare sector, which includes more responsibility in terms of communication, collaboration and patient management. It was quite clearly addressed by the chiropractors that they did not see the profession as united in this issue (difference of opinions), and they did not think that all colleagues delivered the same standard of care. It was clear how the Norwegians, to a larger degree, talked about embracing the role, whereas the Danes were more ambivalent, and the Swedes more hesitant.

It’s still not our second nature to be part of the team now. But we’re still a little like a lonesome cowboy who’s been given a responsibility …but I know of colleagues who thinks of certification rights as a hassle. They would rather treat, examine and treat and continue their workday. But that’s why I think it’s more and more important that you embrace the role as a primary care provider. (NO2-40)

You have to think about how you can make it work, what it is you accept. How do you make it work? That’s the hardest part. And then make sure you have 100% support before you start. Make sure everybody is onboard. Also those who don’t give a damn. (DK3-30)

and

That is how it is. Then, well, I don’t think the limitations are disinterest, I just think we have not been ready for it. (S3-8)

### The social systems that facilitate day-to-day implementation

#### Scope of practice awareness (all)

Public knowledge of chiropractors and their scope of practice were frequently mentioned as barriers in Norway and Sweden.

I think it is missing, in some way, enlightenment about who we are, and where we come from and respect for the education, we actually have. That kind of enlightenment… (S4-8)

I still have patients who doesn’t know that I prescribe sick leave. (NO1-35)

In Denmark, it was also mentioned that the political climate ran counter to changing the system, and that there wasn’t a willingness to provide the necessary funding for collaboration between stakeholders and for holding dialogue meetings.

And in reality that’s where….if you want it to work, then you would need those round table discussions, right?....I don’t know how often they are held, but I don’t think it’s often, and again because it’s so damn expensive. (DK2-8)

#### Patient fee and expectations (Denmark)

The final barriers, mentioned by most of the Danish chiropractors, were the patient fee and patients’ expectations of manual treatment.

…I know that, at least some [patients] think whether they can afford the consultations or find an alternative… (DK4-21)

and

But actually, some patients are not really set on paying for just a piece of advice, they think it’s strange to pay to go for longer walks. (DK2-24)

## Discussion

The practitioners in this study described different levels of integration in their respective healthcare settings and how the legislation impacts the clinical encounter and the level of their involvement in SAM. In Norway, SAM was described as highly integrated in the clinical encounter and as “part of the tool box”, whereas in Denmark the participants described SAM as a pending issue and questioned “Is it worth the trouble?” In Sweden, where chiropractors usually are not part of the national health services, SAM is not integrated in routine practice, and the profession is still struggling to gain broad public recognition. In both Norway and Sweden, the participants described sick leave certification rights as a seal of approval of the profession.

The sickness certification rights in Norway were negotiated as part of a larger agreement regarding reimbursement schemes and referral rights to imaging and medical specialties between the Norwegian Chiropractic Association and the Norwegian government. The sickness certification rights were included in the negotiations as an “expendable” item (personal communication). Much to the surprise of many Norwegian chiropractors, the certification rights were never discussed in detail, but granted without further ado (personal communication). This situation serves as an example of how professional territories are acquired by political manoeuvres rather than by virtue of the content of clinical work [32]. It also shows how the negotiations of this particular role extension were shaped by national policies in relation to public services (i.e. the reimbursement and referral rights). Our interviews reflect that, despite the certification rights in Norway, it is still not clear to all participants whose responsibility it is to assist the patient in a given situation. This is especially evident when talking about SAM of complex cases and cases with psychosocial components. Uncertainty around roles and confusion about accountability between GPs and AHPs are not uncommon in primary care settings [33], and themes from our synthesis appear to echo the general findings from integration experience amongst other healthcare professionals [19, 34].

The legitimacy that sickness certification rights add to the profession was perceived differently in the three countries. Over the past 25 years, the Danish chiropractic community has raised its professional profile to that of an acknowledged contributor to local musculoskeletal healthcare. Thus, it is perhaps the first example of the chiropractic profession being accepted into mainstream healthcare as an equal partner [35]. This was reflected in our findings, where issues of professional legitimacy were raised, predominantly by the Swedish and Norwegian participants. While the Swedish chiropractors struggle to establish legitimacy to integrate fully with established healthcare (primary legitimacy), the Norwegians battle for factors to boost legitimacy to the level of benchmark healthcare professions (secondary legitimacy), e.g., GPs [35].

The rationale of chiropractors as SAM partners put forward by the participants are highly linked to issues of legitimacy. Respondents based their claims on perceptions of their own and other professions’ specialist expertise to justify their part in the certification process and the role extension concept [36]. The chiropractors highlighted their skill in musculoskeletal conditions and alluded to GP’s knowledge gap and lack of time to legitimize role extension claims. In studies of role extension, the use of a common discourse to discredit the competitor profession, either on the basis of their approach to clinical care or their skills or competence is common [19, 34, 36]. As another rationale for chiropractors as SAM partners, the Norwegian and Danish chiropractors argued the need for adoption of a holistic approach towards SAM. They legitimized their role through highlighting their approach of considering “the full picture” and the routine practice of frequent contacts with the patients. In previous studies involving the nursing profession, references to “patient-centred” and “holistic care” were made, clearly as a form of professional rhetoric designed to support their bid for legitimacy in claiming role exclusivity, or at least primacy [19], and similar have been reported in a study of GPs and physiotherapists [36].

We identified a range of barriers for the chiropractors to engage in SAM. Especially salient were organizational or systemic factors, such as the patients’ fee-for-service, which was voiced as a barrier for seeing patients with lower socioeconomic status. Affordability has been identified as a significant gap in health system coverage [37], and it is a problematic barrier in the context of a social welfare system were the pursuit of equality in health including access to healthcare is an overriding goal and principle [38].

Communication was described as an essential part of SAM, but the chiropractors described a predominantly unidirectional flow to other stakeholders and perceived this to be an important barrier for SAM. For the Swedish chiropractors in particular, lack of communication because of the absence of formal communication platforms was especially pertinent. In Denmark, we found that the lack of financial incentives and the administrative burden were important barriers for engaging in SAM. In a systematic review, Kilgour et al. [39] suggested that reduction of organizational pressure and improving communications between stakeholders could ensure that healthcare providers are more amenable to operating in compensation systems. The likely benefit would be a corresponding positive influence on patients’ recovery and return to work [39].

### Methodological considerations and future directions

This study used a qualitative description with in-depth, semi-structured interviews. This provided a broad perspective as well as a deep understanding of the participants’ experiences and perceptions regarding this previously uncharted topic. The study was conducted as part of a mixed-method study, and will inform a quantitative phase. Therefore, we see the present results as a first exploration of SAM among Scandinavian chiropractors. Further interviews may have provided other perspectives. A specific concern is the lack of triangulation with other stakeholders such as GPs, patients or case-workers. In the quantitative phase, we will extend the data collection to representative samples from all three countries.

Healthcare systems, employment settings and work legislation vary considerably internationally, and the findings from this study may not be applicable to settings outside the Scandinavian countries. However, we have found commonalities with existing literature in the area of professional legitimacy and role extension. The chiropractic’s professional development is considered a test case with the potential to affect other AHPs with aspirations to move towards mainstream healthcare [35]. Therefore, the tension between complementary and alternative medicine providers and ordinary healthcare systems, and the Danish chiropractors’ consequent moves toward mainstream inclusion have been observed with interest by contemporary social scientists. [35]. We hypothesize that the legislative rights of sickness certification granted to the Norwegian chiropractors and manual therapists would potentially influence other APHs who work in the area of WDP. Overall, our results support a widely accepted notion that the healthcare division of labour is based not on stationary professional roles, but on dynamic shifts influenced by forces such as the health policy agenda, and may not always favour the traditionally most powerful profession [19].

## Conclusion

AHPs, in this instance chiropractors, with patient management expertise can fulfil a key role in SAM and by extension work disability prevention when these practices are legislatively supported. In cases where these practices occur informally, however, practitioners face systemic-related issues and professional self-image challenges that tend to hamper them in fulfilling a more integrated role as providers of WDP practices.

## Abbreviations

AHP: Allied healthcare provider
CAM: Complementary and alternative medicine
GP: General practitioner
SAM: Sickness absence management
WDP: Work disability prevention
